# Dynamic and Energetic Aspects of Carotenoids In-and-Around Model Lipid Membranes Revealed in Molecular Modelling

**DOI:** 10.3390/ijms25158217

**Published:** 2024-07-27

**Authors:** Marta Pasenkiewicz-Gierula, Jakub Hryc, Michal Markiewicz

**Affiliations:** Department of Computational Biophysics and Bioinformatics, Faculty of Biochemistry, Biophysics and Biotechnology, Jagiellonian University, 30-387 Krakow, Poland; jakub.hryc@doctoral.uj.edu.pl (J.H.); m.markiewicz@uj.edu.pl (M.M.)

**Keywords:** spontaneous membrane intercalation, preferred membrane orientation, free energy gain, timescales, lipid-soluble antioxidant, *trans*-*cis* photoisomerisation

## Abstract

In contrast to plants, humans are unable to synthesise carotenoids and have to obtain them from diet. Carotenoids fulfil several crucial biological functions in the organism; however, due to poor solubility in water, their bioavailability from plant-based food is low. The processes of carotenoid absorption and availability in the human body have been intensively studied. The recent experimental findings concerning these processes are briefly presented in the introductory part of this review, together with a summary of such topics as carotenoid carriers, body transport and tissue delivery, to finally report on molecular-level studies of carotenoid binding by membrane receptors. The main message of the review is contained in the section describing computational investigations of carotenoid intercalation and dynamic behaviour in lipid bilayers. The relevance of these computational studies lies in showing the direct link between the microscopic behaviour of molecules and the characteristics of their macroscopic ensembles. Furthermore, studying the interactions between carotenoids and lipid bilayers, and certainly proteins, on the molecular- and atomic-level using computational methods facilitates the interpretation and explanation of their macroscopic properties and, hopefully, helps to better understand the biological functions of carotenoids.

## 1. Carotenoids: Basic Information

Carotenoids are pigments synthesised predominantly by photosynthetic organisms, i.e., plants, algae and cyanobacteria, but some non-photosynthetic bacteria and fungi can also do so, e.g., [1,2]. In contrast, animals and humans are unable to synthesise carotenoids de novo even though they are critically important to their nutrition and health. In plants, carotenoids play fundamental roles in photosynthesis and photoprotection, e.g., [3,4,5]. In humans, carotenoids are vital in maintaining good health, e.g., as antioxidants, they reduce the risk of various chronic diseases such as cancer and cardiovascular diseases; β- and α-carotenes are precursors of vitamin A, whose deficiency can cause blindness; xanthophylls lutein and zeaxanthin, as macular pigments, decrease the onset of age-related macular degeneration and cataract, e.g., [2,6,7,8,9]. Lutein, as well as zeaxanthin, accumulate in the human brain improve its cognitive functions [10,11,12]. 

**Figure 1:**
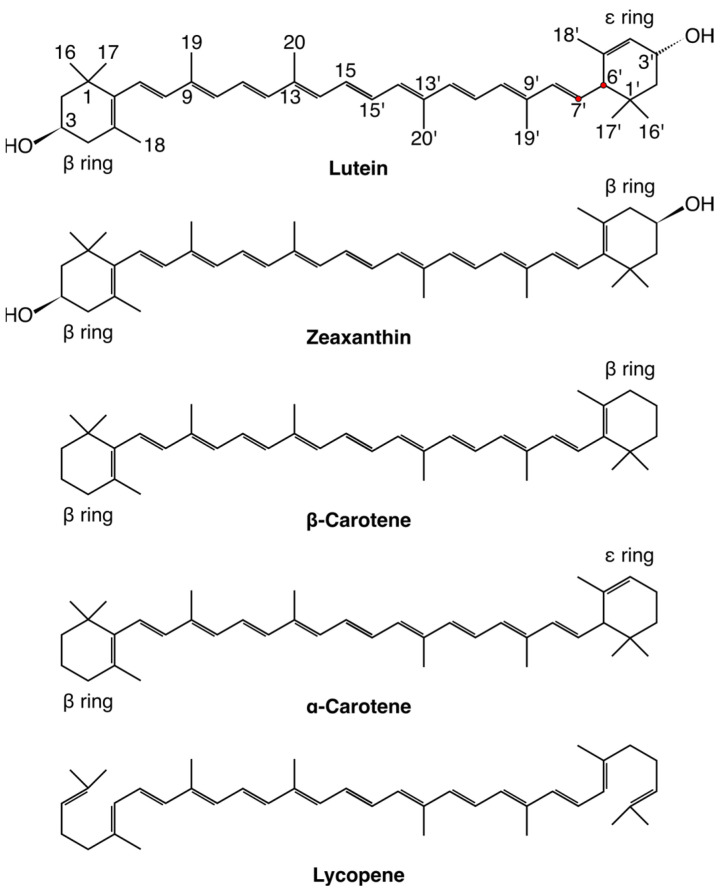
The chemical structures of lutein, zeaxanthin, β-carotene, α-carotene and lycopene. The ionone ring and polyene chain atoms that are used in the text are indicated in lutein only. The atoms are numbered according to the IUPAC convention. The C6′ and C7′ atoms of lutein are indicated with red circles. The chemical symbols for carbon atoms, C and the hydrogen atoms have been omitted except for the OH groups of lutein and zeaxanthin.

At present, 1204 natural carotenoids have been identified from 722 source organisms. Their chemical structures and other information are collected in the Carotenoids Database (http://carotenoiddb.jp, (accessed on 1 July 2024)) established by Yabuzaki [13]. Among the known carotenoids, lycopene, β-carotene (β,β-carotene), α-carotene ((6′R)-β,ε-carotene), lutein and zeaxanthin (Figure 1) are indicated as the most beneficial to humans, e.g., [6,14,15]. The key structural element of all of them is a nearly linear [16] nonpolar polyene chain consisting of eight isoprene units (40-carbon carotenoids) with conjugated double bonds in the *trans* conformation. With the exception of lycopene, these carotenoids have two terminal ionone rings in the C6 and C6′ positions of the polyene chain (Figure 1). In the β- and α-carotene, the rings are unsubstituted, and in lutein and zeaxanthin, they are monohydroxylated at positions C3 and C3′. Thus, lycopene, β- and α-carotene are nonpolar and belong to the group of carotenes, whereas lutein and zeaxanthin are oxygenated derivatives of α- and β-carotene, respectively, and belong to the group of xanthophylls. It is worth noting that both halves (C6–C15 and C15′–C6′, Figure 1) of the polyene chain of the carotenoids are related by 180° rotation about a vertical axis placed at the chain centre (C_2h_ symmetry). Apart from the polyene chain symmetry, lycopene, β-carotene and zeaxanthin are symmetric molecules (C_2h_ symmetry), as both ionone rings of β-carotene and zeaxanthin are the same (β) and lycopene has no terminal rings. In contrast, both α-carotene and lutein have two different rings (β and ε); thus, the molecules are asymmetric. Each of the small differences in the chemical structures of the molecules has its biological relevance and determines carotenoid-protein and carotenoid–lipid bilayer interactions. 

## 2. The Journey of Carotenoids through the Human Body

As was mentioned above, humans are unable to synthesise carotenoids de novo, so they have to obtain them from their diet. The main source of dietary carotenoids are coloured fruits and green leafy vegetables [6]. To become available to the human organism, carotenoids must first be released from the cellular matrix of fruit- and vegetable-based food products. This takes place in the gastrointestinal tract during digestion. As carotenoids are highly nonpolar, to facilitate their absorption in the intestine, they are dispersed into the lipid droplets in the stomach. Then, they are incorporated into bile salt and other lipid mixed micelles in the small intestine [17,18,19,20]. Next, free carotenoids are absorbed by intestinal absorptive cells via scavenger receptors or passive diffusion, incorporated into ultra-low-density lipoproteins (chylomicrons) and secreted into the lymph, e.g., [17,18,19,21,22,23,24]. In general, chylomicrons are taken up by the liver where carotenoids are integrated into either very low-density lipoproteins (VLDL), low-density lipoproteins (LDL) or high-density lipoproteins (HDL) [25,26,27,28] or stored. Roughly, xanthophylls are associated mainly with HDL, and carotenes mainly with LDL and VLDL [23,25,26,28,29], although the exact distribution of carotenoids among lipoproteins is not known for certain [27]. Lipoproteins transport carotenoids in the blood and deliver them to specific tissues for more or less selective uptake.

During their digestion, secretion and delivery, carotenoids exchange, in the free form, between tissues and supramolecular ensembles, which they are temporarily associated with, several times before they reach the sites of their main biological activity. There is a large number of such sites in the human organism; thus, carotenoids are widely distributed among various organs and tissues. However, different tissues accumulate different amounts of carotenoids and for different purposes. For example, the liver collects virtually all carotenoids mainly to transfer them to different lipoproteins and release them into the bloodstream. Adipose tissue accumulates carotenoids rather indiscriminately mainly to store them. In contrast, lutein and zeaxanthin are the only carotenoids that incorporate into the macula lutea, a specialised tissue at the centre of the retina of the human eyes. Additionally, lutein and zeaxanthin are predominant carotenoids in human brain tissue. Carotenoids also accumulate in other tissues, but those mentioned above are the most representative. There are details concerning the tissue distribution of carotenoids and their functions in Refs. [10,11,23,30,31,32], but as pointed out by Landrum [33], carotenoids are rather non-specifically accumulated in tissues, with important exceptions for lycopene in the prostate and lutein and zeaxanthin in the retina.

From a practical perspective, information on how carotenoids are transferred from the plasma lipoproteins to specific tissues is, undoubtedly, the most useful. It would be natural to expect that there are several transfer mechanisms specific to the tissue and the type of carrier. Extensive molecular biology research has revealed that the selective transfer of carotenoid molecules from their carriers to tissues occurs via cell membrane receptors [34]. In the case of macular pigments, intake of zeaxanthin into the retinal pigment epithelia (RPE) cells proceeds via scavenger receptor class B type 1 (abbreviated as SR-B1, SRB1 or SCARB1), which is a multifunctional receptor that binds a broad range of lipoproteins, with a high affinity for HDL [35,36,37]. The structure and the detailed structure-function analysis of the full-length homology model of human SR-B1 predicted by the molecular modelling software package trRosetta [38] are presented in Ref. [39]. However, intake of lutein into the RPE cells proceeds via the LDL receptor (LDLR) [35,36]. The receptor for LDL-bound β-carotene, let alone that for LDL-bound α-carotene, has not yet been identified conclusively, although it is likely that it is LDLR [32,35,40], but SR-B1 has also been considered [32,41]. 

β- and α-carotene are provitamin A carotenoids. In the human intestine, about half of them are converted to vitamin A, and the other half are absorbed intact. In β-carotene conversion, the molecule is cleaved by β-carotene 15,15′-oxygenase 1 at the C15,C15′ double bond into two retinal molecules that are subsequently converted into two retinol (vitamin A) molecules [42]. Cleavage of α-carotene eventually gives one retinol and one α-retinol molecule. In the liver, retinol associates with soluble retinol binding protein 4 (RBP4). The RBP4-retinol complex is released into the bloodstream and delivered to tissues via stimulated by retinoic acid 6 (STRA6) receptor, which transports retinol across the cell membrane [43,44,45]. In peripheral cells, all-*trans*-retinol (vitamin A) is converted into 11-*cis*-retinal, a light-sensitive molecule, and into all-*trans* retinoic acid, a signalling molecule [44]. In the eye, 11-*cis*-retinal is the chromophore of rod and cone opsins. After binding retinal, apo-protein opsin becomes a rod (rhodopsin) or cone (cone opsin) photoreceptor protein. As a signalling molecule, all-*trans* retinoic acid binds to the ligand-binding domains of two retinoic acid nuclear receptors, RAR and RXR, while RAR/RXR heterodimers bind specifically to DNA [44,46].

RBP4 that binds and delivers retinol to specific tissue does not bind α-retinol. Nevertheless, animals fed with α-retinol maintained about 50% of the growth rate of those fed with retinol [47]. This indicates that even though α-retinol has no vitamin A activity, it probably shares the ability of retinoic acid to act as a transcription factor [47]. This also indicates that α-retinol is transported as α-retinyl ester in lipoproteins, as does retinyl ester, which is a storage form of retinol.

## 3. Carotenoid–Receptor Binding: Molecular-Level Studies 

The transfer of carotenoids from plasma lipoproteins to their receptors on the cells of the targeted tissues is a molecular-level process. The methods employed in studying such a process are both experimental and computational. Naturally, molecular-level experiments can provide information with different resolution. Thus, experiments carried out at the macroscopic molecular level have provided physicochemical and biochemical data on carotenoid–protein interactions [27,28,45,48,49,50,51,52,53]. Experiments carried out at the microscopic molecular level, in combination with a computational approach, have provided high-resolution data of the atomic structure of some carotenoid-binding proteins and the location and structure of the carotenoid molecules bound by them [34,50,54,55,56,57,58,59,60]. The results of these and earlier studies have greatly enhanced the knowledge and deepened the understanding of carotenoid absorption, transport and tissue delivery. However, there are still many topics concerning carotenoid binding, transfer and insertion to and between proteins and lipoproteins that have yet to be revealed. It is thus important to study the processes and elucidate their molecular and atomic-level details, as a deeper understanding of them may help in improving the bioavailability of carotenoids in the body [23,61].

## 4. Carotenoids: Transfer from Receptor to Membrane

**Figure 2:**
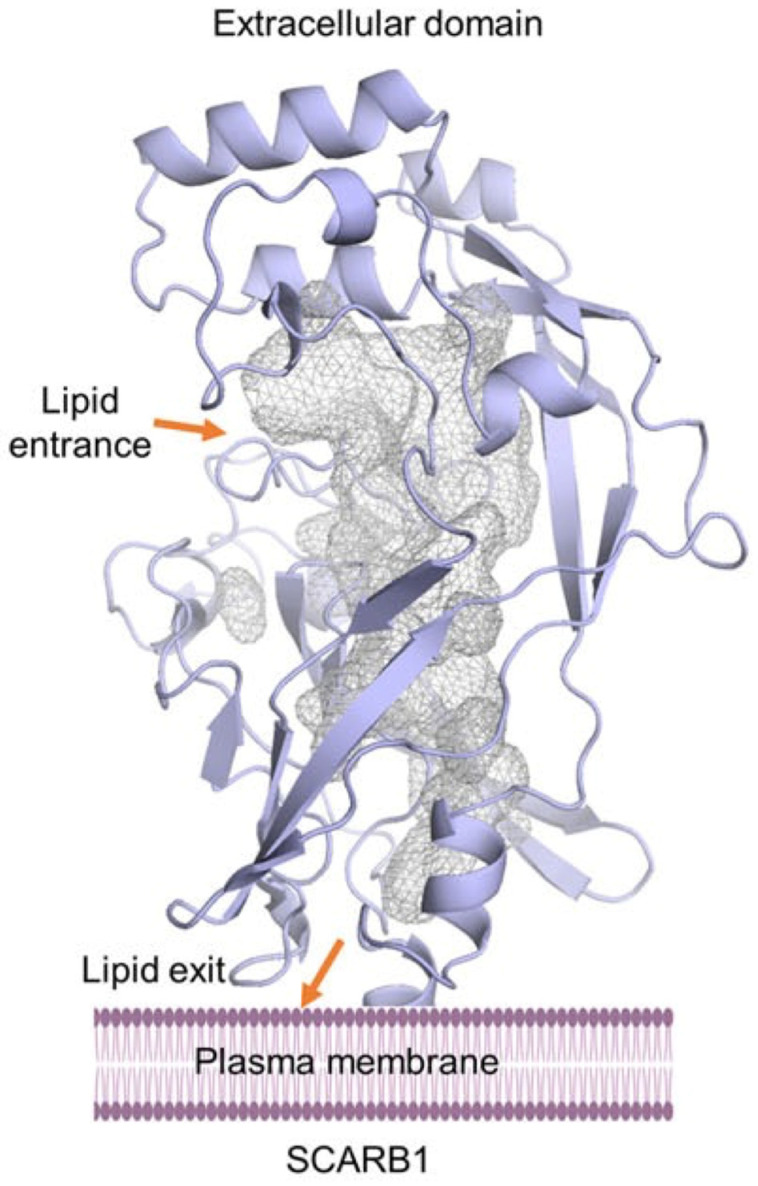
Homology model of class B scavenger receptor. Extracellular domain model of the human SCARB1 (SR-B1) receptor with a proposed lipid entering and exiting tunnel highlighted with a grey colour mesh. Figure. From Bandara, S., & von Lintig, J. (2022). Aster la vista: Unraveling the biochemical basis of carotenoid homeostasis in the human retina. BioEssays, 44, e2200133 [34]. https://doi.org/10.1002/bies.202200133. CC BY 4.0.

In the body, carotenoids are transported in lipoproteins, and from them, they are transferred to the cell through more or less specific membrane-bound receptors, but the ultimate location of biologically active carotenoids in the cell is predominantly the cell membrane. Unfortunately, there is not much well-confirmed information regarding how they arrive there. This is mainly because determining the three-dimensional structures of large multi-domain membrane proteins is a complex and difficult process and not always successful; consequently, none of the structures of human carotenoid receptors have been solved so far. Knowledge of the structure will certainly make predicting how a carotenoid molecule inserts into the membrane feasible. Fortunately, important progress has been made recently as the full-length human SR-B1 receptor has been expressed and purified [53] and, moreover, its 3D structure predicted by homology modelling has been published [39]. The homology model structure of SR-B1 has also been predicted with SWISS-MODEL [62] in Ref. [34] and with MODELLER [63] in Ref. [60]. All three predicted structures have shown that the extracellular domain of SR-B1 has a large, predominantly hydrophobic [60] cavity that serves as a tunnel through which a lipid (carotenoid) passes from the lipoprotein to the plasma membrane. The putative sites where the lipid (carotenoid) enters and exits the tunnel [34] are marked in Figure 2. 

**Figure 3:**
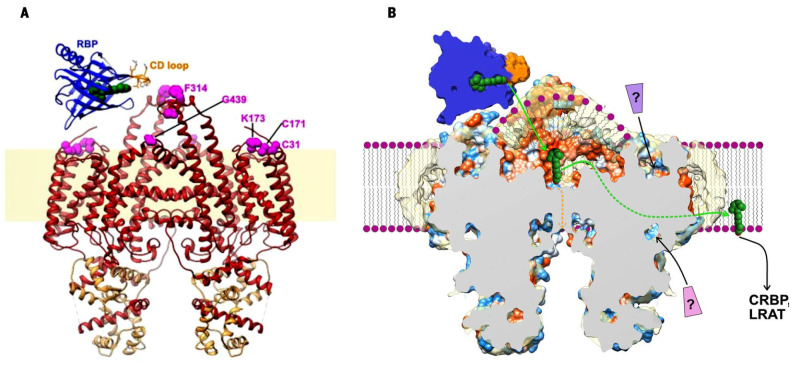
Possible mechanism for STRA6-mediated retinol uptake. (**A**) Ribbon representation of STRA6 (*red* OK ) and RBP (*blue*). Retinol is shown as *green* spheres. The lipid bilayer is shaded light yellow. (**B**) Schematic of STRA6-mediated retinol (*green* spheres) release from RBP into the outer cleft and translocation to the lipid bilayer (shown as *purple* spheres and wavy lines) through the lateral window. Question marks indicate putative ligand binding sites; *green* and *orange* arrows show two potential retinol exit pathways. Figure (modified). From: Yunting Chen et al., Structure of the STRA6 receptor for retinol uptake. *Science* **353**, aad8266 (2016). https://doi.org/10.1126/science.aad8266 [45]. Reprinted with permission from AAAS.

The situation concerning the membrane receptor, STRA6, for retinol binding protein RBP is more fortunate as its dimeric structure has been solved by cryo-electron microscopy [45]. Knowledge of the STRA6 structure has made it possible to indicate the probable routes of retinol transfer from RBP through STRA6 to the membrane [45]. The routes are schematically shown in Figure 3B.

Experimental studies with atomic resolution provide detailed structures of biomolecules but are not able to record their motion simultaneously. This is because they do not possess sufficiently high concurrent spatial and temporal resolution. At present, only computational molecular modelling methods possess such resolution. Unfortunately, computer simulations of the lipid translocation from the donor lipoprotein to the lipid bilayer through the receptor tunnel have not yet been published, so this issue still remains elusive.

## 5. Carotenoids in the Membrane: Atomic-Level Motional Studies 

Classical molecular dynamics (MD) simulation is one of the computational molecular modelling methods. This widely used method has an atomic spatial resolution and sub-picosecond (ps) temporal resolution but has its limitations too. Its main limitations concern the size of the simulated system and the time of its “observation”. Nevertheless, at present, the all-atom MD simulation allows systems to be studied that contain over a million atoms and processes on the µs timescale sampled with 2–4 fs time steps [64]. However, it should be remembered that each model predicted in MD simulations has to be validated through experiments.

There are several computational and experimental papers where the effect of carotenoids on lipid bilayers have been investigated, predicted and reviewed, e.g., [65,66,67,68,69,70]. In general, they have focused on the effect of carotenoids on the lipids in the bilayer and analysed changes in their ordering, orientation and dynamics, as well as on the bilayer physical, mechanical and phase properties. Conversely, here, the main focus is put on a carotenoid molecule in the lipid bilayer and, in particular, on its membrane intercalation and dynamic behaviour. The studies that are reviewed below were performed using computational methods, but the significance of computer predictions is discussed in the light of experimental findings.

The first MD simulation study on the behaviour of carotenoids in lipid bilayers was carried out on a simple model where four β-carotene molecules were inserted into a hydrated palmitoyl-oleoyl-phosphatidylcholine (POPC) bilayer, parallel to the bilayer normal (vertically), in Ref. [71]. During 4 ns MD simulation, the molecules tilted by ~20° and located their rings near the positions of the phospholipid carbonyl groups; the simulation was, though, too short to allow for larger configurational changes in the system. The next MD simulation study focused on spontaneous intercalation into and subsequent orientation of lutein molecules in the POPC bilayer [72]. In each of two bilayer systems, six lutein molecules were placed horizontally on the bilayer surfaces on the side of the hydrating water. In each of them, one molecule intercalated into the bilayer within the first two ns of MD simulation from the β-ring side, and five did not. These ten molecules (five in each system) aggregated in the water, and during 200 ns simulation, they remained there as aggregates. The remaining two lutein molecules intercalated into the upper leaflet of the bilayer, one vertically and the other horizontally. At ~70–80 ns of MD simulations, the molecules changed their positions to horizontal and vertical (transmembrane), respectively, and remained in these positions until the end of the 200 ns simulations. These orientations were stabilised mainly via hydrogen bonds (H-bond) between the lutein OH groups, and, in the case of the vertical orientation, phosphate and carbonyl groups of POPC and water, and in the case of the horizontal orientation, carbonyl groups of POPC and water [72].

The study of lutein intercalation into a POPC bilayer was pursued further in Ref. [73]. In this study, four bilayer systems were built, each containing six lutein molecules. In the initial structures, the luteins were situated almost vertically to the bilayer plane with the “entering ring” of each molecule placed within the regions of the PC phosphate groups and the rest of the molecule immersed in the hydrating water. In two systems, the “entering ring” was the lutein β ring, and in another two, it was the ε ring. The systems were MD simulated for 20–100 ns depending on the time it took for lutein to intercalate fully into the bilayer. Of the 24 lutein molecules present in the systems, 12 either fully or partially spontaneously intercalated in the bilayer, 10 intercalated from the β ring side (~80%) and 2 intercalated from the ε ring side (~17%). The remaining 12 molecules aggregated in the water phase and stayed there as aggregates during the whole simulation time. Five of the intercalated molecules were analysed in detail. Four of them intercalated vertically, and one intercalated horizontally. The time needed by a vertically translocating molecule to reach the interface of the other bilayer side ranged between ~7.5–15 and ~95 ns, both for the β and ε “entering ring”. It is interesting to note that both the β and the ε ring of the lutein that intercalated into the bilayer horizontally were H-bonded with 1–3 water molecules during the whole simulation time. Irrespective of the final orientation of the lutein in the bilayer, both its rings were H-bonded with water and PC polar groups; these interactions stabilised the orientations [73].

To understand the reason for the discrepancy between the number of lutein molecules intercalated with the “entering β ring” and with the “entering ε ring”, the barriers to the β and ε ring lutein intercalation into the bilayer were evaluated from the free energy profiles of transfer of a lutein molecule from the water phase into the bilayer [73]. The profiles were calculated using umbrella sampling simulations [74]. The positions of the barriers to lutein intercalation from the β and the ε ring end coincided with the region of the rigid POPC glycerol groups. The barriers were low, although that encountered by the ε ring was ~2 kcal/mol higher than that encountered by the β ring. The difference in the barrier heights stemmed from different orientations of the β and ε rings relative to the flat polyene chain, which resulted from *sp2* hybridisation of the C6 carbon atom and *sp3* hybridisation of the C6′ carbon atom. When lutein intercalated into the bilayer from the β ring end, the system gained 19.5 ± 1.0 kcal/mol, and from the ε ring end, the gain was 17.9 ± 1.1 kcal/mol [73].

The first MD simulation study where reorientation of carotenoid molecules in the lipid bilayer was analysed in detail was described in Ref. [75]. In this study, either a single β-carotene or zeaxanthin molecule was inserted into a dimyristoyl-PC (DMPC) bilayer either vertically or horizontally, with the latter in the central bilayer region. During 100 ns and 200 ns simulations, the orientation of the β-carotenes varied more than that of the zeaxanthins, but the molecules eventually assumed relatively stable orientations, and the β-carotenes were more tilted than the zeaxanthins. These results were confirmed in umbrella sampling simulations [74], which provided free energy profiles for the rotation of β-carotene and zeaxanthin from the horizontal to the vertical position in the bilayer. The relatively flat profile for β-carotene between 90 and 35° implied that in this range of angles, β-carotene had no preferential orientation, whereas the preferential orientation of zeaxanthin in the bilayer was ~35°. Moreover, the system gained over 3 kcal/mol when zeaxanthin rotated from 90 to 35°. For both carotenoids, orientations between 35 and 0° were energetically unfavoured. Moreover, on the basis of the model that utilised a detailed conformational analysis of the single-bond torsion angles along the conjugated polyene chain of β-carotene and zeaxanthin, the authors estimated the effective length of the chain of the molecules in the bilayer and in water. The transmembrane position of zeaxanthin made its chain more extended, which might have resulted in its enhanced antioxidant capability. However, this was not the case with β-carotene [75]. 

Orientations of xanthophylls in the phospholipid bilayer were also analysed in Ref. [76]. In this study, which was carried out both experimentally and computationally, free energy profiles for rotating zeaxanthin and lutein from the horizontal (90°) to the vertical (10°) position in the DMPC bilayer were calculated using umbrella sampling simulations [74]. The profile for the zeaxanthin was similar to that in Ref. [75]. The minor differences concerned the free energy minimum, which was at ~25° in Ref. [76], and the energy gain due to rotation from 90 to 25°, which was ~4 kcal/mol in Ref. [76]. The free energy profile for rotating lutein in the bilayer showed that the energy gain due to rotation was 3 kcal/mol, thus smaller than that for zeaxanthin, but the free energy minimum was also at ~25°. As their experiment had no means of determining the molecule’s orientation at the free energy minimum, the authors compared the average values of the xanthophyll tilt obtained from unbiased MD simulations and from experimental measurements; the values turned out to be close to each other, which mutually validated the model and the measurements [76]. The values of the average tilt of lutein derived from the experiment and computation were virtually the same as those, respectively, of zeaxanthin. This result was surprising because, as could be anticipated from the difference in the free energy profiles, the horizontal orientation of lutein in the bilayer was ~10 times more probable than that of zeaxanthin [76]. 

**Figure 4:**
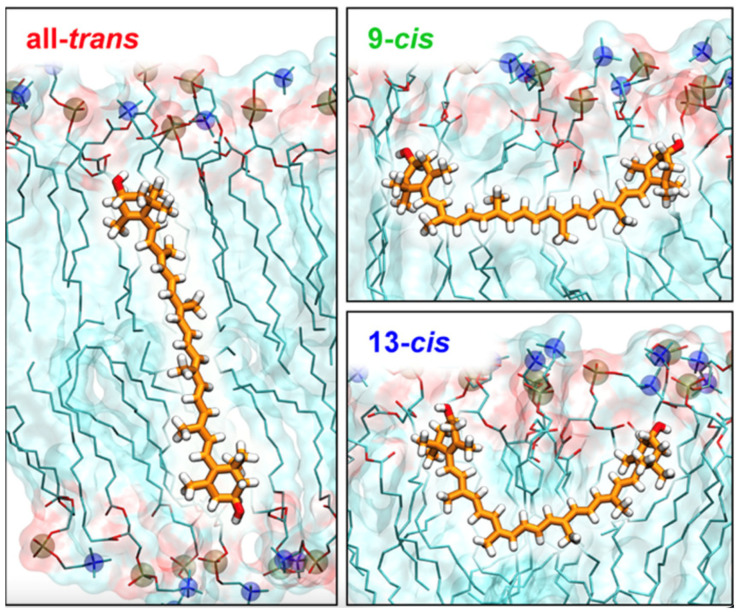
Representative structures for the perpendicular orientation of all-trans zeaxanthin (**left**) and the horizontal orientations of its 9-*cis* and 13-*cis* isomers (**right**) in the DPPC membrane. Figure. Form *J. Phys. Chem. B* 2021, 125, 23, 6090–6102. https://doi.org/10.1021/acs.jpcb.1c01198. [77] CC BY 4.0.

The study carried out in Ref. [77] was a cutting-edge piece of research performed both experimentally and computationally. Its aim was to identify a regulatory mechanism of controlling the intensity of light reaching photoreceptors in the human eye. This molecular-level mechanism, which took place in the retinal cell membranes, was based on the rotation of xanthophyll molecules induced by *trans*-*cis* photoisomerisation. Mutually perpendicular orientations of *trans* and *cis* xanthophyll isomers acted as modulators of light absorption. In the computational part of the research, unbiased MD and umbrella sampling [74] simulations were employed to predict the orientation of zeaxanthin and its two isomers, 9-*cis* and 13-*cis*, in two bilayers. One of them was composed of dipalmitoyl-PC (DPPC), and the other was composed of lipids typical for the retinal cell membrane, distearoyl-PC (DSPC), stearoyl-docosahexaenoyl-phosphatidylethanolamine (SDPE) and SD-phosphatidylserine (SDPS). The simulations confirmed the previous results [76] that *trans* zeaxanthin in the bilayer was oriented vertically, whereas 9-*cis* and 13-*cis* zeaxanthin were in 2 and 45%, respectively, of cases oriented horizontally (Figure 4). Both the simulations and the experimental measurements identified the same molecular mechanisms of controlling the intensity of light reaching photoreceptors in the retina [77].

Orientations of echinenone (β,β-caroten-4-one, 4-keto-β-carotene) and β-carotene in the lipid bilayer were investigated in Ref. [78]. In this study, a mixed-lipid bilayer made of PC and PE with 18- and 16-carbon atom acyl chains of varying unsaturation that represented lipid composition of natural egg yolk, was used. The bilayer containing one molecule of echinenone or β-carotene was MD simulated for 1 µs. The results showed that although β-carotene could reorient in the bilayer rather freely, it resided most often (78% of the simulation time) in the middle of one of the bilayer leaflets oriented almost horizontally, at 80–85°, with the bilayer normal. Less often (22% of the simulation time), β-carotene was oriented 40–45° relative to the bilayer normal. Echinenone in the bilayer was in a transmembrane position with a preferential tilt at ~30°. The results for β-carotene were in general agreement with those in Ref. [75], and the orientation and position of echinenone in the bilayer were similar to those of zeaxanthin in Ref. [76]. However, detailed comparison is not fully justified, as in both studies, related but not the same quantities were analysed, and the bilayers used had different lipid compositions.

In Ref. [79], orientations of β-carotene and zeaxanthin in six mixed-lipid bilayers in different phases were compared. The bilayers were composed of POPC, DSPC and cholesterol in different proportions such that some bilayers were in the liquid-disordered and some in the liquid-ordered phase. In each phase, the distribution of the β-carotene orientations was broader than that of zeaxanthin. Moreover, in each bilayer, the carotenoids caused bilayer thinning. This result was at variance with the result in Ref. [80], which showed that both β-carotene and zeaxanthin increased the POPC bilayer thickness. The contradicting results might have stemmed from different lipid compositions of bilayers used in both studies, and possibly from too short an equilibration time of the bilayers in Ref. [79]. 

**Figure 5:**
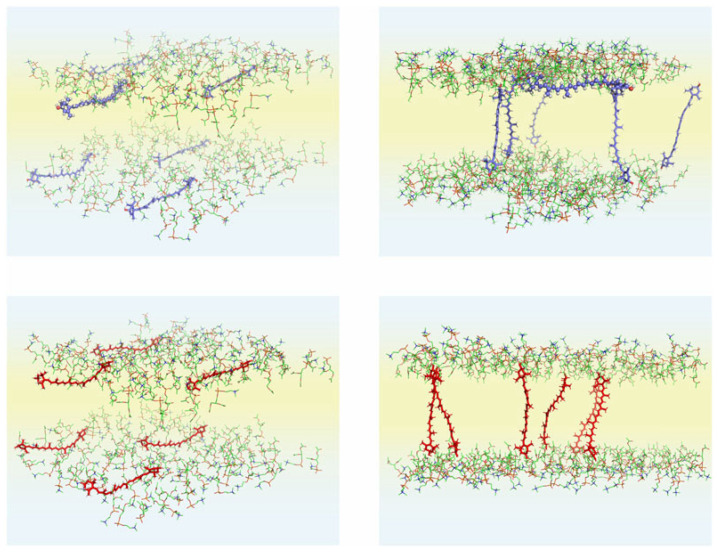
Initial (**left** column) and final (**right** column; after 1100 ns of MD simulations) structures of the POPC bilayer containing lutein (**upper** row) and zeaxanthin (**lower** row). In the initial structure, six xanthophyll molecules were placed parallel to the bilayer surface (horizontally). The lutein molecule, which remained in the horizontal position during the whole simulation time, is presented in the ball-and-stick model. In the figures, only the POPC head groups and xanthophyll molecules are shown to better illustrate the details. The atoms are represented in standard colours, except for the xanthophyll carbon atoms, which are *blue* for lutein and *red* for zeaxanthin. Water is shaded *light blue,* and the lipid nonpolar region is shaded *light yellow*. Figure. Form Makuch K, Hryc J, Markiewicz M and Pasenkiewicz-Gierula M (2021) Lutein and Zeaxanthin in the Lipid Bilayer–Similarities and Differences Revealed by Computational Studies. *Front. Mol. Biosci*. 8:768449. https://doi.org/10.3389/fmolb.2021.768449. [81] CC-BY.

The MD simulations described in Ref. [81] were carried out to investigate the reorientational dynamics and preferred orientation of two xanthophylls in four POPC bilayers on the µs timescale. In the initial structures, six molecules of lutein or zeaxanthin were placed in the bilayer either vertically or horizontally. The molecules oriented horizontally were located in the region between POPC glycerol and phosphate groups (Figure 5, left-hand side); thus, no molecules aggregated in the water phase. Each system was MD simulated for 1.1 µs. The molecules could reorient freely, although none of the vertically placed xanthophyll molecules reoriented to the horizontal position. In contrast, all horizontally placed zeaxanthin molecules and five out of six horizontally placed lutein molecules reoriented to the vertical position during MD simulation times ranging from 10 to 500 ns. One lutein molecule remained in the horizontal position for the whole MD simulation time [81] (Figure 5, upper right). 

To answer the question as to why none of the zeaxanthin molecules remained in the horizontal orientation, whereas one out of six lutein molecules did remain so, detailed conformational analyses of the C5-C6-C7-C8 (β ring) and C5′-C6′-C7′-C8′ (ε ring) torsion angles (called β-ring torsion and ε-ring torsion, respectively) were performed. The β-ring torsion of lutein and zeaxanthin in vacuum, water and the bilayer had two low-energy conformations that were close to each other, of 30° and −30°, and separated by a relatively low energy barrier; therefore, transitions between them were very frequent. The ε-ring torsion of lutein in vacuum had two low-energy conformations of 130° and −50°. Even though the angular distance between them was quite large and they were separated by a relatively high barrier, transitions between them were moderately frequent. However, for lutein in water and in the bilayer, the torsion occupied only the lower-energy conformation of 130°. H-bonds of lutein OH groups with polar groups of water and PC, together with a relatively high-energy barrier, hindered transitions between the conformational states. Moreover, analyses of the interactions between water molecules and the methyl groups of the polyene chain, as well as the OH groups of the ionone rings, of horizontally oriented lutein and zeaxanthin revealed which interactions with water and which orientations of the rings played a role in stabilising the horizontal orientation of the molecules in the bilayer. The result that the ε-ring torsion of lutein in the bilayer occupied only one conformation was contrary to a commonly made claim that “free” rotation of the ε ring about the single C6′–C7′ bond is the possible cause of the horizontal location of lutein in the bilayer. Conversely, the fixed conformation of the ε-ring torsion, the mutually perpendicular orientations of the lutein ε and β rings (the β ring is coplanar with the polyene chain plane, the ε ring is nearly perpendicular to it) and the orientation of the β ring OH group of the horizontal lutein were the key factors that made a horizontal lutein less likely to rotate to the vertical position than a horizontal zeaxanthin [81]. Of note was the observation that the plane of the polyene chain of any molecule that entered the bilayer from the horizontal orientation was perpendicular to the bilayer surface. 

The phospholipid bilayers used in the MD simulation studies described above were, broadly speaking, simple models of an unspecified animal cell membrane. In Ref. [82], the behaviour of two carotenoids, lycopene and zeaxanthin, in a computer model of the human *stratum corneum* (SC) cell membrane was investigated. In human skin, as in other organs, carotenoids play a protective role against photodamage [83]. The main lipid species of the lipid matrix of the SC cell membrane is ceramides with acyl chains of different lengths. In this study, the lipid bilayer was built of ceramide NS24 (a sphingolipid with a 24-carbon-atom fatty acid linked via an amide). The bilayer with one inserted molecule of lycopene or zeaxanthin was MD simulated for 100 ns. The preferred orientation of zeaxanthin in the ceramide bilayer was similar to that in the PC bilayer [75,76,77,81], i.e., vertical [82]. In contrast, the preferred orientation of lycopene was horizontal. The results of unbiased MD simulations of bilayers with a single carotenoid molecule were confirmed by umbrella sampling simulations [74]. The free energy profile for rotating zeaxanthin from 90 to 0° indicated that its preferred orientation in the bilayer was ~25°, and that due to rotation, the system gained over 3 kcal/mol; these results conform well to those in Refs. [75,76,77], even though the bilayers used in those studies had different lipid compositions. Rotating lycopene from 90 to 0° resulted in an almost monotonic increase in the system free energy, which indicated that the preferred orientation of lycopene in the bilayer was ~90°, i.e., horizontal [82].

MD simulation of β-carotene in a bilayer made of lipids typical for the brain, POPC and DMPS, was carried out in Ref. [84]. Details concerning the simulation system were nevertheless scarce; the study revealed that even though (an unspecified number of) β-carotene molecules located initially in the water hydrating the bilayer aggregated, they, over time, spontaneously intercalated into the bilayer as an aggregate. As can be seen in the video attached to Ref. [84], a β-carotene molecule that intercalated into the bilayer at the beginning of the simulation as a single species, during a (probably) 200 ns MD simulation, had considerable rotational freedom there but was in the vertical position quite often. Once the aggregate intercalated into the bilayer, the single molecule joined it. 

A combined experimental and theoretical study of the aggregation of carotenoids in the bilayer was carried out in Ref. [85]. Experiments that employed several methods were performed on chiral carotenoids that differed in polarity, i.e., α-carotene (nonpolar), zeaxanthin and fucoxanthin (polar), in the DPPC bilayer. MD simulations and quantum-chemical calculations were made only on an α-carotene monomer and dimer inserted horizontally into the middle of the DPPC bilayer. Both in a monomeric and a dimeric state, the molecules moved about and rotated in the bilayer core rather freely; thus, the “dimer” they formed was not firm, and their tilts relative to the bilayer normal were broadly spread, between 30 and 140°, with an average of 100°. Even though the molecules in the “dimer” were, on average, ~21 Å apart from each other, they somehow affected each other, e.g., they became more bent than as monomers. The difference in the bending between monomeric and dimeric α-carotene expressed in Å was very small, although it was quite apparent in QM calculations. The stabilising effect of bending on the “dimer” structure shown in simulation and confirmed experimentally indicated the propensity of α-carotene to aggregate in the bilayer in contrast to more polar and vertically oriented zeaxanthin and fucoxanthin [85]. 

## 6. Carotenoids in the Membrane: A Summary of Computational Studies and Their Significance

What have we learnt from computer modelling studies about the behaviour of 40-carbon carotenoids near and in a lipid bilayer? Starting from the beginning: When carotenoid molecules are put into water hydrating the bilayer, some of them aggregate there, but some spontaneously intercalate into the bilayer. Both outcomes are due to their long nonpolar polyene chains. Unfortunately, simulations do not conclusively resolve whether the molecules intercalate the bilayer as monomers or as larger aggregates. While in some simulations, intercalation is monomeric, some others have indicated that multimers can also intercalate. In an MD simulation study [86], lutein intercalated into the POPC bilayer both as a monomer and a dimer, although in the subsequent MD simulations, the latter never happened [72,73,81]. A large β-carotene aggregate intercalating into the POPC-DMPS bilayer was shown in Ref. [84]. An experiment, in contrast to a simulation, has no means to tell what the multimeric form of carotenoids intercalating the bilayer is, but it is able to discriminate between carotenoid monomers and multimers in the bilayer as well as the type of aggregate formed, e.g., [85,87,88]. α-Carotene self-assembled readily in the gel-phase DPPC bilayer and formed “head-to-tail” J-aggregates of uncertain sizes that remained stable when temperature was increased [85]. Carotenoids with polar groups, namely fucoxanthin, zeaxanthin and lutein, formed “side-by-side” H-aggregates of rather small sizes were unstable at elevated temperatures, and in liquid-phase bilayers, they were in the monomeric form [85,87,88]. This experimental result conforms to the results of 1.1-μs MD simulations of zeaxanthin and lutein in the POPC bilayer at 310 K where the molecules did not show any sign of aggregation [81]. 

Spontaneity of xanthophyll intercalation into the lipid bilayer directly stems from the negative value of the change in free energy for the process. The calculated free energy gain in lutein (zeaxanthin) intercalating the bilayer is ~20 kcal/mol [73]. MD simulations and free energy calculations have also demonstrated that the intercalation of lutein from the β ring end is more probable than from the ε ring end; the barrier for the ε ring to pass through the PC glycerol groups region of the bilayer was ~2 kcal/mol higher than that for the β ring [73]. 

MD simulations have confirmed experimental results that the vertical position of xanthophyll molecules in the bilayer is far more probable than the horizontal one, whereas umbrella sampling simulations have given numerical values of the free energy gain due to the rotation of lutein and zeaxanthin from the horizontal to the vertical positions; the values were in the range <3, 5> kcal/mol [75,76,77,82]. The gain, which is altogether not very large, is smaller for lutein than zeaxanthin, which makes the horizontal orientation of lutein in the bilayer ~10 times more probable than that of zeaxanthin [76]. These energetical evaluations are in harmony with the results of MD simulations in Ref. [81], where out of 12 xanthophyll molecules placed horizontally in the bilayer, 11 spontaneously reoriented to vertical position, and the molecule that did not reorient was lutein. In contrast to polar, nonpolar carotenoids, β-carotene, α-carotene and lycopene are mainly in the horizontal position in the lipid bilayer; this is evident from experimental measurements, MD simulations [75,78,82,85] and free energy calculations [75,82]. Echinenone, with only one polar group, is in the PC-PE bilayer [78], orientated similarly to that of zeaxanthin in the PC bilayer [75].

The answer to the question as to why the vertical or horizontal orientation of carotenoids in the membrane is important can provide only experimental or quantum mechanical studies. Here, one can only speculate, bearing in mind that the main function of carotenoids is to protect the cell from the damaging effects of light and oxygen. Let us take macular pigments as an example. The maximum absorption of light by macular pigments is at 460 nm, the wavelength corresponding to blue light, which is damaging to retinal cells. The cornea and lens of the eye absorb 99.34% of incident UV light, so in practice, it does not reach the retina [89]. Macular pigments absorb ~60% of the incident blue light at 460 nm and 40–46% of the entire damaging wavelength range from 400 to 500 nm [90,91]. The highest concentration of macular pigments is in the photoreceptor axon layer, which precedes the photoreceptors; macular pigments thus act as an effective pre-receptoral light filter [91,92]. The transmembrane (vertical) position of lutein and zeaxanthin makes it possible to fulfil their task. However, the task would presumably be accomplished more effectively if the xanthophylls were in the horizontal position, which would allow them to absorb blue light from all directions [93,94]. MD simulations have indicated that this might be the case only for lutein and probably on a very short timescale [73,81]. However, recent results of the Gruszecki group [77] demonstrated that light-induced *cis*-isomerisation of xanthophylls in human retina samples and in model membranes triggered their reorientation from a vertical to a horizontal position. The consequences of the reorientation are briefly discussed above and in detail in Refs. [77,95]. 

Blue light that reaches the retina may initiate photosensitised production of singlet oxygen. Thus, xanthophylls, as pre-receptoral light filters, act as passive antioxidants [33,92]. They are also active antioxidants as they are able to quench singlet oxygen and other reactive oxygen species [33,92,94,95,96]. However, the ability of macular pigments to protect the retina stems mainly from their selective location in the domains of retinal cell membranes that are enriched in phospholipids with long polyunsaturated acyl chains, which are especially vulnerable to oxygen damage. In these domains, they act as a lipid-soluble antioxidant, and their vertical as well as horizontal position there plays a crucial role in phospholipid protection [95,97,98,99,100]. 

Computer simulations supplement experimental data not only by providing numerical values for the free energy gain on xanthophyll intercalation or rotation in the membrane, but also by predicting the timescales of the processes. Full intercalation of a lutein molecule into the POPC bilayer is very fast and ranges between ~7.5–15 and ~95 ns for either β or ε ring intercalation [73]. The same time range applies to zeaxanthin. The predicted time of lutein and zeaxanthin reorientation from the horizontal to the vertical position is in the range from 10 to 500 ns [81]. One lutein molecule did not reorient during the simulation time of 1.1 µs in Ref. [81]. In classical MD simulation, such an observation time is considered rather long, but it is very short compared to that of experimental measurements on macroscopic samples. As lutein in a horizontal position has not been observed experimentally [77], it is quite probable that in a longer MD simulation, the horizontal lutein reorients to the vertical position. However, one should then recall that photochemical reactions are very fast. Photoexcitation of lutein populates its second singlet excited state, S_2_, that decays in a few hundred femtoseconds to the lowest singlet excited state, S_1_, which then relaxes back to the ground state, S_0_, on a picosecond timescale [101,102]. Thus, during the lifetime of its horizontal orientation (>1 µs), lutein may participate in many light-generated processes. Moreover, even though the horizontal position is much less probable than the vertical one, both orientations must be in some statistical equilibrium in the bilayer.

This review clearly shows that a combined experimental and computational approach in studying molecular-level biological processes provides us with the opportunity to know and understand them better. It also shows that our knowledge concerning how carotenoids are transported in the body and enter the cell membrane is rather fragmentary. However, hopefully, this review might help the reader in recognising topics that merit more in-depth study. 

## References

[1] Fuentes P., Pizarro L., Moreno J.C., Handford M., Rodriguez-Concepcion M., Stange C. (2012). Light-dependent changes in plastid differentiation influence carotenoid gene expression and accumulation in carrot roots. Plant Mol. Biol..

[2] Sun T.H., Rao S.M., Zhou X.S., Li L. (2022). Plant carotenoids: Recent advances and future perspectives. Mol. Hortic..

[3] Gómez-Sagasti M.T., López-Pozo M., Artetxe U., Becerril J.M., Hernández A., García-Plazaola J.I., Esteban R. (2023). Carotenoids and their derivatives: A “Swiss Army knife-like” multifunctional tool for fine-tuning plant-environment interactions. Environ. Exp. Bot..

[4] Demmig-Adams B., Stewart J.J., López-Pozo M., Polutchko S.K., Adams W.W. (2020). Zeaxanthin, a molecule for photoprotection in many different environments. Molecules.

[5] Caferri R., Guardini Z., Bassi R., Dall’Osto L., Wurtzel T.E. (2022). Assessing photoprotective functions of carotenoids in photosynthetic systems of plants and green algae. Methods Enzymol.

[6] Eggersdorfer M., Wyss A. (2018). Carotenoids in human nutrition and health. Arch. Biochem. Biophys..

[7] Milani A., Basirnejad M., Shahbazi S., Bolhassani A. (2017). Carotenoids: Biochemistry, pharmacology and treatment. Br. J. Pharmacol..

[8] Fiedor J., Burda K. (2014). Potential role of carotenoids as antioxidants in human health and disease. Nutrients.

[9] Zhao Z.L., Chen J., Ci F.F., Pang H., Cheng N., Xing A.J. (2022). α-Carotene: A valuable carotenoid in biological and medical research. J. Sci. Food Agric..

[10] Stringham J.M., Johnson E.J., Hammond B.R. (2019). Lutein across the Lifespan: From Childhood Cognitive Performance to the Aging Eye and Brain. Curr. Dev. Nutr..

[11] Lopresti A.L., Smith S.J., Drummond P.D. (2022). The Effects of Lutein and Zeaxanthin Supplementation on Cognitive Function in Adults With Self-Reported Mild Cognitive Complaints: A Randomized, Double-Blind, Placebo-Controlled Study. Front. Nutr..

[12] Parekh R., Hammond B.R., Chandradhara D. (2024). Lutein and zeaxanthin supplementation improves dynamic visual and cognitive performance in children: A randomized, double-blind, parallel, placebo-controlled study. Adv. Ther..

[13] Yabuzaki J. (2017). Carotenoids Database: Structures, chemical fingerprints and distribution among organisms. Database-Oxford.

[14] Amengual J. (2019). Bioactive Properties of Carotenoids in Human Health. Nutrients.

[15] Pitts S.B.J., Johnson N.S., Wu Q., Firnhaber G.C., Kaur A.P., Obasohan J. (2022). A meta-analysis of studies examining associations between resonance Raman spectroscopy-assessed skin carotenoids and plasma carotenoids among adults and children. Nutr. Rev..

[16] Fiedor L., Dudkowiak A., Pilch M. (2019). The origin of the dark S state in carotenoids: A comprehensive model. J. R. Soc. Interface.

[17] Harrison E.H. (2012). Mechanisms involved in the intestinal absorption of dietary vitamin A and provitamin A carotenoids. Bba-Mol. Cell Biol. Lipids.

[18] Lemmens L., Colle I., Van Buggenhout S., Palmero P., Van Loey A., Hendrickx M. (2014). Carotenoid bioaccessibility in fruit- and vegetable-based food products as affected by product (micro) structural characteristics and the presence of lipids: A review. Trends Food Sci. Tech..

[19] Xavier A.A.O., Mercadante A.Z. (2019). The bioaccessibility of carotenoids impacts the design of functional foods. Curr. Opin. Food Sci..

[20] Parker R.S. (1996). Carotenoids.4. Absorption, metabolism, and transport of carotenoids. FASEB J..

[21] Chacón-Ordóñez T., Carle R., Schweiggert R. (2019). Bioaccessibility of carotenoids from plant and animal foods. J. Sci. Food Agric..

[22] Reboul E. (2023). Proteins involved in fat-soluble vitamin and carotenoid transport across the intestinal cells: New insights from the past decade. Prog. Lipid Res..

[23] Furr H.C., Clark R.M., Krinsky N.I., Mayne S.T., Sies H. (2004). Transport, uptake, and target. Tissue storage of carotenoids. Carotenoids in Health and Disease.

[24] Gugliucci A. (2024). The chylomicron saga: Time to focus on postprandial metabolism. Front. Endocrinol..

[25] Shmarakov I.O., Yuen J.J., Blaner W.S., Tanumihardjo S.A. (2013). Carotenoid Metabolism and Enzymology. Carotenoids and Human Health.

[26] Canene-Adams K., Erdman J.W., Britton G., Liaaen-Jensen S., Pfander H. (2009). Absorption, transport, distribution in tissues and bioavailability. Carotenoids, Nutrition and Health.

[27] Li B.X., Vachali P., Chang F.Y., Gorusupudi A., Arunkumar R., Shi L.J., Rognon G.T., Frederick J.M., Bernstein P.S. (2022). HDL is the primary transporter for carotenoids from liver to retinal pigment epithelium in transgenic ApoA-I/Bco2 mice. Arch. Biochem. Biophys..

[28] Arunkumar R., Gorusupudi A., Bernstein P.S. (2020). The macular carotenoids: A biochemical overview. Bba-Mol. Cell Biol. Lipids.

[29] Krinsky N.I., Cornwell D.G., Oncley J.L. (1958). The transport of vitamin A and carotenoids in human plasma. Arch. Biochem. Biophys..

[30] Böhm V., Lietz G., Olmedilla-Alonso B., Phelan D., Reboul E., Bánati D., Borel P., Corte-Real J., de Lera A.R., Desmarchelier C. (2021). From carotenoid intake to carotenoid blood and tissue concentrations—Implications for dietary intake recommendations. Nutr. Rev..

[31] Elvira-Torales L.I., García-Alonso J., Periago-Castón M.J. (2019). Nutritional Importance of Carotenoids and Their Effect on Liver Health: A Review. Antioxidants-Basel.

[32] Bohn T., Desmarchelier C., El S.N., Keijer J., van Schothorst E., Rühl R., Borel P. (2019). β-Carotene in the human body: Metabolic bioactivation pathways—From digestion to tissue distribution and excretion. P. Nutr. Soc..

[33] Landrum J.T., Tanumihardjo S.A. (2013). Reactive oxygen and nitrogen species in biological systems: Reactions and regulation by carotenoids. Carotenoids and Human Health.

[34] Bandara S., von Lintig J. (2022). Aster la vista: Unraveling the biochemical basis of carotenoid homeostasis in the human retina. Bioessays.

[35] Thomas S.E., Harrison E.H. (2016). Mechanisms of selective delivery of xanthophylls to retinal pigment epithelial cells by human lipoproteins. J. Lipid Res..

[36] Harrison E.H. (2019). Mechanisms of transport and delivery of vitamin A and carotenoids to the retinal pigment epithelium. Mol. Nutr. Food Res..

[37] Shen W.J., Asthana S., Kraemer F.B., Azhar S. (2018). Scavenger receptor B type 1: Expression, molecular regulation, and cholesterol transport function. J. Lipid Res..

[38] Yang J.Y., Anishchenko I., Park H., Peng Z.L., Ovchinnikov S., Baker D. (2020). Improved protein structure prediction using predicted interresidue orientations. Proc. Natl. Acad. Sci. USA.

[39] Powers H.R., Sahoo D. (2022). SR-B1’s next top model: Structural perspectives on the functions of the HDL receptor. Curr. Atheroscler. Rep..

[40] Quadro L., Giordano E., Costabile B.K., Nargis T., Iqbal J., Kim Y., Wassef L., Hussain M.M. (2020). Interplay between β-carotene and lipoprotein metabolism at the maternal-fetal barrier. Bba-Mol. Cell Biol. Lipids.

[41] Borel P., Moussa M., Reboul E., Lyan B., Defoort C., Vincent-Baudry S., Maillot M., Gastaldi M., Darmon M., Portugal H. (2007). Human plasma levels of vitamin E and Carotenoids are associated with genetic polymorphisms in genes involved in lipid metabolism. J. Nutr..

[42] Reboul E. (2019). Mechanisms of carotenoid intestinal absorption: Where do we stand?. Nutrients.

[43] Steinhoff J.S., Lass A., Schupp M. (2021). Biological functions of RBP4 and its relevance for human diseases. Front. Physiol..

[44] Ask N.M., Leung M., Radhakrishnan R., Lobo G.P. (2021). Vitamin A transporters in visual function: A mini review on membrane receptors for dietary vitamin A uptake, storage, and transport to the eye. Nutrients.

[45] Chen Y., Clarke O.B., Kim J., Stowe S., Kim Y.K., Assur Z., Cavalier M., Godoy-Ruiz R., von Alpen D.C., Manzini C. (2016). Structure of the STRA6 receptor for retinol uptake. Science.

[46] Petkovich M., Chambon P. (2022). Retinoic acid receptors at 35 years. J. Mol. Endocrinol..

[47] Riabroy N., Dever J.T., Tanumihardjo S.A. (2014). α-Retinol and 3,4-didehydroretinol support growth in rats when fed at equimolar amounts and α-retinol is not toxic after repeated administration of large doses. Br. J. Nutr..

[48] Likkei K., Moldenhauer M., Tavraz N.N., Maksimov E.G., Sluchanko N.N., Friedrich T. (2024). Lipid composition and properties affect protein-mediated carotenoid uptake efficiency from membranes. Biochim. Biophys. Acta-Biomem..

[49] Shyam R., Vachali P., Gorusupudi A., Nelson K., Bernstein P.S. (2017). All three human scavenger receptor class B proteins can bind and transport all three macular xanthophyll carotenoids. Arch. Biochem. Biophys..

[50] Bandara S., Ramkumar S., Imanishi S., Thomas L.D., Sawant O.B., Imanishi Y., von Lintig J. (2022). Aster proteins mediate carotenoid transport in mammalian cells. Proc. Natl. Acad. Sci. USA.

[51] Slonimskiy Y.B., Egorkin N.A., Friedrich T., Maksimov E.G., Sluchanko N.N. (2022). Microalgal protein AstaP is a potent carotenoid solubilizer and delivery module with a broad carotenoid binding repertoire. Febs J..

[52] Bandara S., Moon J., Ramkumar S., von Lintig J. (2023). ASTER-B regulates mitochondrial carotenoid transport and homeostasis. J. Lipid Res..

[53] Powers H.R., Jenjak S.E., Volkman B.F., Sahoo D. (2023). Development and validation of a purification system for functional full-length human SR-B1 and CD36. J. Biol. Chem..

[54] Sluchanko N.N., Slonimskiy Y.B., Egorkin N.A., Varfolomeeva L.A., Kleymenov S.Y., Minyaev M.E., Faletrov Y.V., Moysenovich A.M., Parshina E.Y., Friedrich T. (2022). Structural basis for the carotenoid binding and transport function of a START domain. Structure.

[55] Kornilov F.D., Slonimskiy Y.B., Lunegova D.A., Egorkin N.A., Savitskaya A.G., Kleymenov S.Y., Maksimov E.G., Goncharuk S.A., Mineev K.S., Sluchanko N.N. (2023). Structural basis for the ligand promiscuity of the neofunctionalized, carotenoid-binding fasciclin domain protein AstaP. Commun. Biol..

[56] Horvath M.P., George E.W., Tran Q.T., Baumgardner K., Zharov G., Lee S., Sharifzadeh H., Shihab S., Mattinson T., Li B.X. (2016). Structure of the lutein-binding domain of human StARD3 at 1.74 angstrom resolution and model of a complex with lutein. Acta Crystallogr. F.

[57] Shyam R., Gorusupudi A., Nelson K., Horvath M.P., Bernstein P.S. (2017). RPE65 has an additional function as the lutein to meso-zeaxanthin isomerase in the vertebrate eye. Proc. Natl. Acad. Sci. USA.

[58] Varfolomeeva L.A., Slonimskiy Y.B., Egorkin N.A., Minyaev M.E., Faletrov Y.V., Boyko K.M., Sluchanko N.N. (2022). Preparation and structural studies of the silkworm carotenoid-binding protein complexed with a new pigment. Crystallogr. Rep..

[59] Hazai E., Bikádi Z., Zsila F., Lockwood S.F. (2006). Molecular modeling of the non-covalent binding of the dietary tomato carotenoids lycopene and lycophyll, and selected oxidative metabolites with 5-lipoxygenase. Bioorgan Med. Chem..

[60] Neculai D., Schwake M., Ravichandran M., Zunke F., Collins R.F., Peters J., Neculai M., Plumb J., Loppnau P., Pizarro J.C. (2013). Structure of LIMP-2 provides functional insights with implications for SR-BI and CD36. Nature.

[61] Molteni C., La Motta C., Valoppi F. (2022). Improving the bioaccessibility and bioavailability of carotenoids by means of nanostructured delivery systems: A comprehensive review. Antioxidants-Basel.

[62] Waterhouse A., Bertoni M., Bienert S., Studer G., Tauriello G., Gumienny R., Heer F.T., de Beer T.A.P., Rempfer C., Bordoli L. (2018). SWISS-MODEL: Homology modelling of protein structures and complexes. Nucleic Acids Res..

[63] Fiser A., Sali A. (2003). Modeller: Generation and refinement of homology-based protein structure models. Methods Enzymol..

[64] Rizzuti B. (2022). Molecular simulations of proteins: From simplified physical interactions to complex biological phenomena. Biochim. Biophys. Acta Proteins Proteom..

[65] Popova A.V., Andreeva A.S., Iglič A., Genova J. (2013). Chapter Eight—Carotenoid–lipid interactions. Advances in Planar Lipid Bilayers and Liposomes.

[66] Augustynska D., Jemiola-Rzeminska M., Burda K., Strzalka K. (2015). Influence of polar and nonpolar carotenoids on structural and adhesive properties of model membranes. Chem-Biol. Interact..

[67] Cvetkovic D., Fiedor L., Wisniewska-Becker A., Markovic D. (2013). Organization of carotenoids in models of biological membranes: Current status of knowledge and research. Curr. Anal. Chem..

[68] Perez-Lopez M.I., Mendez-Reina R., Trier S., Herrfurth C., Feussner I., Bernal A., Forero-Shelton M., Leidy C. (2019). Variations in carotenoid content and acyl chain composition in exponential, stationary and biofilm states of Staphylococcus aureus, and their influence on membrane biophysical properties. Bba-Biomembranes.

[69] Bykowski M., Mazur R., Wójtowicz J., Suski S., Garstka M., Mostowska A., Kowalewska L. (2021). Too rigid to fold: Carotenoid-dependent decrease in thylakoid fluidity hampers the formation of chloroplast grana. Plant Physiol..

[70] Manrique-Moreno M., Jemiota-Rzeminska M., Múnera-Jaramillo J., López G.D., Suesca E., Leidy C., Strzalka K. (2022). Carotenoids modulate the thermotropic phase behavior of model systems that mimic its membrane composition. Membranes.

[71] Jemiola-Rzeminska M., Pasenkiewicz-Gierula M., Strzalka K. (2005). The behaviour of β-carotene in the phosphatidylcholine bilayer as revealed by a molecular simulation study. Chem. Phys. Lipids.

[72] Pasenkiewicz-Gierula M., Baczynski K., Murzyn K., Markiewicz M. (2012). Orientation of lutein in a lipid bilayer—Revisited. Acta Biochim. Pol..

[73] Makuch K., Markiewicz M., Pasenkiewicz-Gierula M. (2019). Asymmetric spontaneous intercalation of lutein into a phospholipid bilayer, a computational study. Comput. Struct. Biotec.

[74] Torrie G.M., Valleau J.P. (1977). Nonphysical sampling distributions in Monte Carlo free-energy estimation: Umbrella sampling. J. Comput. Phys..

[75] Cerezo J., Zuniga J., Bastida A., Requena A., Ceron-Carrasco J.P. (2013). Conformational changes of beta-carotene and zeaxanthin immersed in a model membrane through atomistic molecular dynamics simulations. Phys. Chem. Chem. Phys..

[76] Grudzinski W., Nierzwicki L., Welc R., Reszczynska E., Luchowski R., Czub J., Gruszecki W.I. (2017). Localization and orientation of xanthophylls in a lipid bilayer. Sci. Rep..

[77] Luchowski R., Grudzinski W., Welc R., Pinto M.M.M., Sek A., Ostrowski J., Nierzwicki L., Chodnicki P., Wieczor M., Sowinski K. (2021). Light-modulated sunscreen mechanism in the retina of the human eye. J. Phys. Chem. B.

[78] Semenov A.N., Gvozdev D.A., Zlenko D.V., Protasova E.A., Khashimova A.R., Parshina E.Y., Baizhumanov A.A., Lotosh N.Y., Kim E.E., Kononevich Y.N. (2022). Modulation of Membrane Microviscosity by Protein-Mediated Carotenoid Delivery as Revealed by Time-Resolved Fluorescence Anisotropy. Membranes.

[79] Johnson Q.R., Mostofian B., Gomez G.F., Smith J.C., Cheng X.L. (2018). Effects of carotenoids on lipid bilayers. Phys. Chem. Chem. Phys..

[80] Mostofian B., Johnson Q.R., Smith J.C., Cheng X.L. (2020). Carotenoids promote lateral packing and condensation of lipid membranes. Phys. Chem. Chem. Phys..

[81] Makuch K., Hryc J., Markiewicz M., Pasenkiewicz-Gierula M. (2021). Lutein and zeaxanthin in the lipid bilayer-similarities and differences revealed by computational studies. Front. Mol. Biosci..

[82] Ri J.S., Choe C.S., Choe S.H., Jong K.H., Hong S.N., Schleusener J., Lademann J., Darvin M.E. (2023). Lycopene, but not zeaxanthin, serves as a skeleton for the formation of an orthorhombic organization of intercellular lipids within the lamellae in the stratum corneum: Molecular dynamics simulations of the hydrated ceramide NS bilayer model. Biochim. Biophys. Acta-Biomem..

[83] Flieger J., Raszewska-Famielec M., Radzikowska-Büchner E., Flieger W. (2024). Skin protection by carotenoid pigments. Int. J. Mol. Sci..

[84] Gastaldo I.P., Himbert S., Ram U., Rheinstädter M.C. (2020). The effects of resveratrol, caffeine, β-carotene, and epigallocatechin gallate (EGCG) on amyloid-β aggregation in aynthetic brain membranes. Mol. Nutr. Food Res..

[85] Hachlica N., Stefanska M., Mach M., Kowalska M., Wydro P., Domagala A., Kessler J., Zajac G., Kaczor A. (2024). Organization of carotenoid aggregates in membranes studied selectively using resonance Raman optical activity. Small.

[86] Pasenkiewicz-Gierula M., Markiewicz M. Orientation of lutein in a lipid bilayer—Revisited. Proceedings of the 16th International Symposium on Carotenoids.

[87] Sujak A., Okulski W., Gruszecki W.I. (2000). Organisation of xanthophyll pigments lutein and zeaxanthin in lipid membranes formed with dipalmitoylphosphatidylcholine. Biochim. Biophys. Acta-Biomem..

[88] Sujak A., Mazurek P., Gruszecki W.I. (2002). Xanthophyll pigments lutein and zeaxanthin in lipid multibilayers formed with dimyristoylphosphatidylcholine. J. Photochem. Photobiol. B.

[89] Fukuoka H., Gali H.E., Bu J.J., Sella R., Afshari N.A. (2023). Ultraviolet light exposure and its penetrance through the eye in a porcine model. Int. J. Ophthalmol-Chi.

[90] Snodderly D.M., Auran J.D., Delori F.C. (1984). The macular pigment. 2. Spatial-distribution in primate retinas. Invest. Ophth Vis. Sci..

[91] Schalch W., Landrum J.T., Bone R.A., Britton G., Liaaen-Jensen S., Pfande H. (2009). The Eye. Carotenoids, Nutrition and Health.

[92] O’Connell E., Neelam K., Nolan J., Au Eong K.G., Beatty S. (2006). Macular carotenoids and age-related maculopathy. Ann. Acad. Med. Singap..

[93] Ahmed S., Lott M., Marcus D. (2005). The macular xantophyls. Surv. Ophthalmol..

[94] Vishwanathan R., Johnson E.J., Tanumihardjo S.A. (2013). Lutein and Zeaxanthin and Eye Disease. Carotenoids and Human Health.

[95] Welc-Stanowska R., Pietras R., Mielecki B., Sarewicz M., Luchowski R., Widomska J., Grudzinski W., Osyczka A., Gruszecki W.I. (2023). How do xanthophylls protect lipid membranes from oxidative damage?. J. Phys. Chem. Lett..

[96] Cantrell A., McGarvey D.J., Truscott T.G., Rancan F., Böhm F. (2003). Singlet oxygen quenching by dietary carotenoids in a model membrane environment. Arch. Biochem. Biophys..

[97] Subczynski W.K., Wisniewska A., Widomska J. (2010). Location of macular xanthophylls in the most vulnerable regions of photoreceptor outer-segment membranes. Arch. Biochem. Biophys..

[98] Widomska J., Gruszecki W.I., Subczynski W.K. (2021). Factors differentiating the antioxidant activity of macular xanthophylls in the human eye retina. Antioxidants.

[99] Widomska J., Zareba M., Subczynski W.K. (2016). Can xanthophyll-membrane interactions explain their selective presence in the retina and brain?. Foods.

[100] Wisniewska A., Subczynski W.K. (2006). Distribution of macular xanthophylls between domains in a model of photoreceptor outer segment membranes. Free Radic. Biol. Med..

[101] Polívka T., Sundström V. (2004). Ultrafast dynamics of carotenoid excited states -: From solution to natural and artificial systems. Chem. Rev..

[102] Accomasso D., Arslancan S., Cupellini L., Granucci G., Mennucci B. (2022). Ultrafast Excited-State Dynamics of Carotenoids and the Role of the S State. J. Phys. Chem. Lett..

